# Are the Results of the Bayley Scales of Infant and Toddler Development (Third Edition) Predictive for Later Motor Skills and School Performance?

**DOI:** 10.3390/children11121486

**Published:** 2024-12-06

**Authors:** Sophia Maria Kipping, Wieland Kiess, Juliane Ludwig, Christof Meigen, Tanja Poulain

**Affiliations:** 1 LIFE Leipzig Research Center for Civilization Diseases, Leipzig University, Philipp-Rosenthal-Strasse 27, 04103 Leipzig, Germany; wieland.kiess@medizin.uni-leipzig.de (W.K.); juliane.ludwig@medizin.uni-leipzig.de (J.L.); christof.meigen@medizin.uni-leipzig.de (C.M.); tanja.poulain@medizin.uni-leipzig.de (T.P.); 2 Department of Women and Children’s Health, Hospital for Children and Adolescents and Center for Pediatric Research (CPL), Leipzig University, Liebigstrasse 20a, 04103 Leipzig, Germany

**Keywords:** Bayley-III, predictive validity, motor skills, cognitive skills

## Abstract

Background/Objectives: The first year of life represents a critical developmental stage in which the foundations for motor, cognitive, language, and social–emotional development are set. During this time, development occurs rapidly, making early detection of developmental disorders essential for timely intervention. The Bayley Scales of Infant and Toddler Development—Third Edition (Bayley-III) is an effective tool for assessing language, motor, and cognitive development in children aged 1 to 42 months. This study aimed to investigate whether or not the results of the Bayley-III in healthy one-year-old children are predictive for their later motor skills and school performance. Methods: This study had a prospective, longitudinal design. The study participants were healthy children having performed Bayley-III at 1 year with information on motor performance (*n* = 170) at age 5–10 and school grades (*n* = 69) at age 7–10. Linear or logistic regression analysis was performed for data analysis. Results: Below-average performance in the cognitive part of the Bayley-III at age 1 was significantly associated with poorer performance in balancing backwards (b = −0.45), sideways jumping (b = −0.42), standing long jump (b = −0.54), and forward bends (b = −0.59) at age 5–10 (all *p* < 0.05). Performance in other parts of the Bayley-III was not significantly associated with later motor skills. Furthermore, we did not observe any significant associations between performance in the Bayley-III and grades in school. The associations were not moderated by age, sex, or socioeconomic status (all *p* > 0.05). Conclusions: The cognitive scale of the Bayley-III may be used as a predictive tool for later motor skills. Regarding school performance, the Bayley-III cannot be considered predictive.

## 1. Introduction

Early childhood represents a critical developmental stage in which the foundations for motor, cognitive, language, and social–emotional development are set [1]. By the age of one, children begin to develop basic cognitive skills, such as grasping object permanence and recognizing familiar people and objects [2]. They also start to understand cause and effect [2]. In language development, they can follow simple commands, say their first words, and use gestures to communicate [2]. Motor development at one year of age includes milestones such as crawling, standing with support, and taking their first steps [2]. Children also begin to refine fine motor skills, such as the pincer grasp [2].

During the first years of life, developmental and learning processes progress at their fastest rates [1,3]. At the same time, delays observed at this early stage might be a sign of developmental disorders that can affect further development [1,3]. Therefore, early detection of developmental disorders is essential to provide affected children with early intervention, leading to the possibility of improved development and functioning [4,5]. To identify these children, the Bayley Scales of Infant and Toddler Development—Third Edition (Bayley-III) can be used [6]. This is a pediatric developmental assessment tool that evaluates the language (expressive and receptive), motor (fine and gross motor skills), and cognitive development of children aged 1 to 42 months [6,7].

There is currently only a limited number of studies on the predictive validity of Bayley-III test results for later motor and cognitive abilities, and the findings from these studies are partly contradictory. Moreover, most studies focus on premature infants. Therefore, further research is needed to assess the predictive validity of Bayley-III in healthy, full-term newborns. This allows for a comprehensive evaluation of early childhood development, contributing to an improved understanding of both typical and atypical developmental trajectories [8].

Klein-Radukic and Zmyj [9] found positive predictive relationships between cognitive performance in the Bayley-III (at first, second and third year of life) and later intelligence quotient (IQ) (at 4 years) in children born at term. Bode et al. [10] examined the predictive validity of the Bayley-III cognitive and language scores in 2-year-old children (former premature infants and a socioeconomically matched control group) for the IQ of these children at preschool age (4 years) and found positive associations. In contrast, Spencer-Smith et al. [11] assessed the Bayley-III cognition and language scores in 2-year-old premature children and examined their predictive power for future developmental disorders (at the age of 4). They rated them as poor predictors. A study by Månsson et al. [8] assessed the relationship between Bayley-III test results (cognitive, language, and motor scales) at the age of 2.5 years and IQ at school age (6.5 years) in full-term newborns with high socioeconomic status (SES). In this study, the cognitive score of Bayley-III was the best predictor for IQ score variability, but at the individual level, Bayley-III was considered an insufficient predictor for later IQ at school age [8]. In a systemic review by Griffiths et al. [12], the Bayley-III demonstrated predictive validity for later gross motor performance, with the highest predictive validity at the age of 2 years. Burakevych et al. [13] rated the motor scores of Bayley-III as poor predictors for later motor skills (compared at 2 and 4.5 years). Similarly, Spittle et al. [14] demonstrated that the motor scores of Bayley-III assessed at 2 years of age underestimated later motor impairments (at 4 years) in prematurely born children.

The present study aimed to determine whether or not the Bayley-III results in one-year-old children born at term predict their later motor skills and school performance. Additionally, we explored the potential moderating effect of sociodemographic factors (age, sex, and SES) on these associations. We hypothesized that we would find a significant positive association between the Bayley-III test results and later school performance, as well as between the Bayley-III test results and subsequent motor performance. These relationships were expected to be more pronounced in vulnerable children (those with lower SES) compared to children with higher SES. We had no specific hypothesis regarding the moderating effect of sex.

## 2. Materials and Methods

### 2.1. Participants

Data were taken from the LIFE Child study, which has been conducted since 2011 as part of the Leipzig Research Center for Civilization diseases (LIFE) at Leipzig University [15]. The LIFE Child study is a prospective, longitudinal cohort study examining child development from the prenatal period to early adulthood [14]. The study participants do not have any chronic, chromosomal, or syndromic conditions [16]. Most of them are from Leipzig or the surrounding area [16]. The study program includes clinical examinations, questionnaires, tests, and the collection of various biological materials at different time points [16].

The LIFE Child study was designed in accordance with the declaration of Helsinki [17] and the study program was approved by the Ethics Committee of the University of Leipzig (Reg. No. 477/19-ek) [14]. The parents sign a fully informed and written consent at each study visit [15].

**Figure 1:**
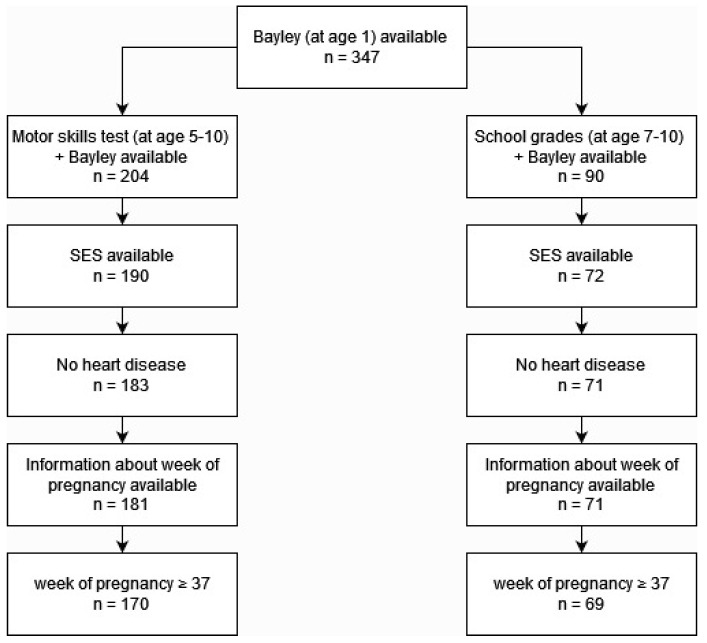
Flowchart of data cleansing for sample 1 (left) and sample 2 (right).

For the present study, all children who had completed the Bayley-III test at the age of 1 year (t1) and, additionally, participated in a motor skills test at age 5 to 10 years (sample 1) and/or provided information on school grades at age 7 to 10 years (sample 2) (t2) were eligible for analysis. In cases where children had participated in a motor skills test several times or had provided information on school grades at several time points, only the last visit was taken into account. Subjects with missing information on SES or week of pregnancy at birth were not considered. Furthermore, children born preterm (<37th week of pregnancy) or having a heart disease were excluded. The individual steps of data cleansing for both samples can be seen in the following flowchart (Figure 1).

After data cleaning, sample 1 comprised 170 participants (55% male, mean age at t1 = 1.0, sd = 0.11; mean age at t2 = 6.4, sd = 0.64) and sample 2 included 69 participants (59% male, mean age at t1 = 1.0, sd = 0.13; mean age at t2 = 8.9 years, sd = 0.72).

### 2.2. Instruments

#### 2.2.1. Bayley-III: Bayley Scales of Infant and Toddler Development (Third Edition)

The Bayley-III is a pediatric developmental testing procedure used for the early detection of developmental delays [18]. It is the internationally best established test for assessing the development of young children [19].

In the context of the LIFE Child study, the third edition of the test (Bayley-III) was used for data collection. It was released in 2006 in the United States. Norms for the German version (released in 2014) were created in 2011 with the help of the LIFE Child study [16]. The German version of the Bayley-III was shown to be a valid and reliable instrument [2]. It includes scales for cognitive, language (expressive and receptive), and motor (fine and gross motor) development for children aged 1 to 42 months [7,18]. The scores are transferred to age-specific standard values (mean = 100, sd = 15). Based on these standard values, performance values in the different domains of the Bayley-III are categorized as either ‘normal to above average’ (cutoff > 85) or ‘below average’ (cutoff ≤ 85). Thus, ‘normal to above average’ was chosen as the reference level. Dichotomizing the Bayley-III results simplifies clinical interpretation by categorizing them into clear groups, aiding in clinical decision-making and interventions.

#### 2.2.2. Motor Skills Tests

As part of the LIFE Child study, the motor skills of the children are measured using a standardized motor skills test [20,21]. The test consists of five parts: balancing backwards, sideways jumping, standing long jump, pushups, and forward bends, which measure children’s coordination, strength, and mobility [22].

In the balancing task, participants walk backward on beams with widths of 6 cm, 4.5 cm, and 3 cm. Each beam includes one test trial forward and one backward, followed by two scoring attempts. A maximum of 8 steps can be scored per attempt, and the trial ends if the participant loses balance or falls off the beam. The sideways jumping task involves the participant jumping with both feet across the centerline of the test area and back as many times as possible within 15 s. Two attempts are made, with a 1 min break between them. The long jump involves the participant jumping from a standing position with slightly bent knees, using arm swing for momentum. Both takeoff and landing must be with both feet. The test is performed twice. The pushup task begins with the participant lying on their stomach with their hands resting on their buttocks. After the start command, they push up to a standard position and return to the starting position. The participant has 40 s to complete as many pushups as possible. In the forward bend task, the participant stands barefoot on a wooden bench with a vertical scale, bending forward with straight knees and reaching as far as possible with outstretched arms. The maximum reach is held for two seconds, and the value is recorded, followed by a brief pause before repeating [22].

The performance in each part was transformed to standard deviation scores (SDSs) (mean = 0, sd = 1) based on sex- and age-specific percentiles assessed in a large representative German sample [23]. Results of Shapiro–Wilks tests showed that all SDSs (with the exception of sideways jumping) were normally distributed (*p* > 0.05). For sideways jumping, a histogram showed a distribution that was very close to a normal distribution.

#### 2.2.3. School Performance

In the LIFE Child study, school performance is measured by grades in the subjects of Mathematics, German, and Physical Education [22]. The information is provided by the parents or self-reported by the children [22]. In Germany, grades vary between 1 (best) and 6 (worst). In the present data set, no participant reported grades 5 or 6. For the analyses, grades were dichotomized into ‘high performance’ (grade 1) and ‘low performance’ (grades 2, 3, and 4) to ensure that the group sizes would be comparable. Even if grade 2 does not indicate poor performance, the term ‘low performance’ was used in this context for better readability.

#### 2.2.4. Socioeconomic Status (SES)

The socioeconomic status was determined as a multidimensional index (SES index) combining information on parental education, profession, and net equivalent income [24]. SES scores ranging from 3 to 21 were categorized as low, medium, and high, based on cut-offs defined after examining a representative German sample [24]. Due to the low percentage of children from families with a low SES (3% in sample 1 and 2), we combined the ‘low’ and ‘medium’ groups to ensure comparable group sizes; i.e., the SES was dichotomized into ‘low/medium’ (*n* = 97 (57%) in sample 1 and 37 (54%) in sample 2) and ‘high’ (*n* = 73 (43%) in sample 1 and 32 (46%) in sample 2).

### 2.3. Statistical Analysis

Data were described in means ± standard deviations (for continuous variables) or numbers/percentages (for categorical variables).

Linear regression analysis was applied to assess associations between cognitive, language, and motor skills in early childhood and motor skills (sample 1) in later childhood. For analyzing the associations between cognitive, language, and motor skills in early childhood and school performance (sample 2) in later childhood, logistic regression analysis was used.

Age in later childhood (at time of the motor skills test or assessment of school performance), sex (male/female), and family SES in early childhood were included as covariates. We also checked whether the associations between early development and later motor skills and school performance were moderated by these covariates. Strengths of associations were represented by non-standardized regression coefficients (sample 1) or odds ratios (sample 2). Interactions with the covariates (moderator analysis) were only presented if they were statistically significant (*p* < 0.05). For the statistical analysis, the program R was used (version R 4.2.2.) [25].

## 3. Results

### 3.1. Performance in Bayley-III, Motor Skills Test, and School Grades

Table 1 summarizes the descriptive statistics for categorical and numerical variables in both study samples. In sample 1, 150, 137, and 137 children had completed the cognitive, language, and motor part of the Bayley-III, respectively. Of these children, 78% (*n* = 117), 69% (*n* = 95), and 78% (*n* = 107) showed ‘normal to above average’ performance in the cognitive, language, or motor part, respectively. Consequently, 22% (*n* = 33), 31% (*n* = 42), and 22% (*n* = 30) showed ‘below average’ performance in the respective parts. The mean percentile rank ± sd for performance in the Bayley-III were 97.13 ± 14.81 for the cognitive part, 92.3 ± 17.42 for the language part, and 96.84 ± 14.03 for the motor part. The average SDSs for performance in the motor skills test were 0.05 ± 1.1 for balancing backwards, −0.35 ± 1.0 for sideways jumping, 0.03 ± 1.01 for standing long jump, −0.01 ± 1.04 for pushups, and −0.15 ± 1.22 for forward bends.

In sample 2, 63, 55, and 50 children had completed the cognitive, language, and motor part of the Bayley-III, respectively. Of these children, 83% (*n* = 52), 60% (*n* = 33), and 82% (*n* = 41) showed ‘normal to above average’ performance in the cognitive, language, or motor part, respectively. Consequently, 17% (*n* = 11), 40% (*n* = 22), and 18% (*n* = 9) showed ‘below average’ performance in the respective parts. The mean percentile rank ± sd for performance in the Bayley-III was 98.81 ± 13.25 for the cognitive part, 91 ± 17.01 for the language part, and 97.5 ± 14.59 for the motor part. Regarding school performance, 41% (*n* = 28) had a ‘high’ and 59% (*n* = 41) had a ‘low’ grade in Mathematics. For German, the distribution was 29% (*n* = 20) ‘high’ and 71% (*n* = 49) ‘low’. In Physical Education, 22% (*n* = 15) showed ‘high’ and 78% (*n* = 54) ‘low’ performance. The average SDSs for the school grades were 1.68 ± 0.65 for Mathematics, 1.82 ± 0.62 for German, and 1.56 ± 0.5 for Physical Education.

### 3.2. Associations Between Bayley-III Results and Later Motor Skills

**Figure 2:**
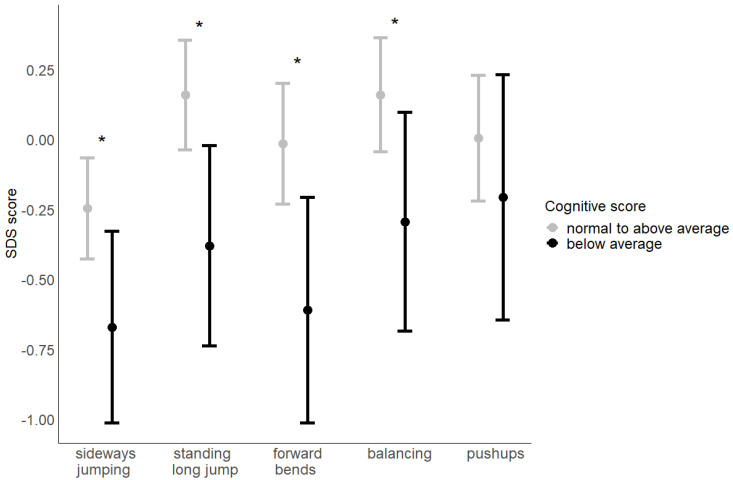
Estimated mean performance (+95% confidence interval) in the different parts of the motor skills test at age 5–10 years in children who showed normal to above average or below average performance in the cognitive part of the Bayley-III at age 1 year. * *p* ≤ 0.05.

A below-average performance in the cognitive part of the Bayley-III was significantly associated with poorer performance in balancing backwards (b = −0.45, *p* = 0.045), sideways jumping (b = −0.42, *p* = 0.033), standing long jump (b = −0.54, *p* = 0.010), and forward bends (b = −0.59, *p* = 0.012). In more detail, the motor skill performance of children who showed a below-average performance in the cognitive part of the Bayley-III at the age of one year was about half a standard deviation lower than the motor performance of children who showed an average or above-average performance. These associations are also illustrated in Figure 2. Performance in the other parts of the Bayley-III were not significantly associated with later motor skills (see Table 2). The moderator analysis showed that the associations were not significantly moderated by age, sex, or SES (all *p* > 0.05).

### 3.3. Associations Between Bayley-III Results and Later School Grades

With respect to school performance, we did not observe any significant associations between performance in the Bayley-III scales at one year of age and grades in school at age 7–10 (see Table 3). The moderator analysis showed that the associations were not significantly moderated by age, sex, or SES (all *p* > 0.05).

## 4. Discussion

### 4.1. General Discussion

The present study assessed 1-year-old children’s performance in the Bayley-III and investigated its predictive validity for later motor skills and school performance. Understanding this relationship is of clinical significance because it facilitates the identification of necessary support and intervention, empowering informed decision-making for both parents and professionals. Regarding the Bayley-III results, the amount of below-average performance in the motor and cognitive parts (approximately 20%) was slightly higher than expected (in a representative sample, only 15% should score below average). In the language part, an especially large proportion of children performed below average (30–40%). Since the language part was performed after the cognition and sometimes even after the motor skills part, this finding might be explained by concentration and motivation difficulties. In general, conducting the Bayley-III requires a high level of examination effort and a long-lasting concentration ability of the children [26]. This concentration level is influenced by many factors, such as sleep, hunger, time of day, and mood [27], and might decrease with increasing time of assessment.

Regarding motor skills at age 5–10, the average performances in the different parts of the test lay in the expected range (SDS −1 to +1). With respect to school grades at age 7–10, however, we observed a strong tendency towards very good grades. This might be explained by the high SES of participating families.

### 4.2. Predictive Validity of Bayley-III for Later Motor Skills

The analyses of the present study revealed significant associations between a below-average performance in the cognitive part of the Bayley-III and poorer performance in the motor skills test. These results are comparable with the systematic review of Griffiths et al. [12] stating a good predictive validity of the Bayley-III at the age of 2 years for future movement abilities (gross motor assessment). Cognitive and motor abilities are interconnected and follow a similar temporal development, which progresses most rapidly during kindergarten and elementary school years [28,29]. If there is a restriction in cognition, e.g., due to a neurological condition, this often affects both cognitive and motor functions [30]. Conversely, in case of a motor function disorder, such as a developmental coordination disorder, cognition is typically altered as well [30]. This can be explained by co-activations between the prefrontal cortex, the cerebellum, and the basal ganglia during various motor and cognitive tasks [30]. Peyre et al. [31] investigated whether motor development in the preschool period can be predicted by prior performance in other cognitive domains (language, attention, emotion, behavioral, and socialization skills) and, overall, the study concluded that children’s cognitive capabilities are predictive for motor characteristics [31]. Child age or sex, and the family’s SES did not moderate the observed associations between Bayley-III results and later motor skills, indicating that the strengths of these associations are not affected by these socio-demographic factors.

Interestingly, performance in the motor part of the Bayley-III was not significantly related to later motor skills. This is in line with previous studies that rated the motor scores of Bayley-III as poor predictors of later motor skills [13,14]. Possible reasons for this finding are that the motor difficulties occur only in later childhood, that motor abilities show a strong fluctuation, and that the Bayley-III might not be the best test to evaluate proficient motor skills [13]. The tasks of the motor part of the Bayley-III and the motor skills task might be too different. The fine motor subscale of the Bayley-III encompasses grip development, sensorimotor integration, and fine motor action planning and speed, and the gross motor subscale assesses motor skills of the limbs and trunk, such as static postural control, movement control, locomotion, balance, and gross motor action planning [2]. Thus, the Bayley-III motor score may assess functions that contribute to general development rather than specific motor functions [13]. The motor skills test, in contrast, captures very specific motor skills [20].

### 4.3. Predictive Validity of Bayley-III for Later School Performance

We did not observe any significant associations between the test results of the Bayley-III and later school performance. The associations between performance in the cognitive or language part of the Bayley-III and later school performance pointed in the expected direction, while the association between early motor skills and later school performance did not. To the best of our knowledge, other studies in this context mainly focused on the predictive validity for later cognitive skills by using intelligence assessments. According to Duggan et al. [32], for example, the Bayley-III has poor predictive validity for cognitive skills at school age. The authors concluded that the Bayley-III can predict a normal performance but that children with low cognitive skills at school age might not be detected. Further studies showed similar results [8,12,33,34]. In contrast, other researchers rated the Bayley-III as a significant predictor for later IQ [11] or later cognitive delay [35]. Comparisons should be made with caution as school grades and IQ are not the same. School grades are not only affected by a child’s IQ [36] but also by several other factors including self-regulation [37,38], SES [36], family size [36], or physical fitness [29]. The high number of potential influencing factors shows the dynamic nature of the academic development of primary school children, which could explain the missing associations in our study. In this context, the ongoing debate regarding the psychometric properties of grades is noteworthy [39]. While some argue that grades are mainly relevant for university admissions and lack significance beyond academics, others highlight their predictive value for accessing higher education and their link to developmental outcomes in young adulthood [39].

According to Rubio-Codina and Grantham-McGregor [40], the predictive validity of the Bayley-III increases with age at which the Bayley-III is performed. We assessed the Bayley-III at the age of 1 year, which could also be an explanation for the missing association with later school performance. Additionally, the time range between the Bayley-III and the school performance might have been too long. Finally, the size of this sample was very small. In small samples, only very strong associations can be detected/reach statistical significance.

### 4.4. Strengths and Limitations

We compared the Bayley-III at the age of 1 year with a motor skills test between the ages of 5 and 10 years and school grades between the ages of 7 and 10 years. Therefore, we were able to look at a large age range and, thus, to analyze a longer-term predictive validity than in previous studies. Further, as far as we know, no previous study examined the relationship between Bayley-III results and school grades.

One limitation of this study is its restricted representativeness, as the cohort exhibits a trend towards a higher SES [15]. Further, the small sample size is a limiting factor, through which small and medium effect sizes might not have been detected. Additionally, the dichotomization of school grades (1 vs. 2–4) represents a restriction of our study. In general, the psychometric properties of school grades and the extent of their predictive validity require critical consideration. Furthermore, the wide age ranges investigated (5–10 and 7–10 years) encompass significant developmental periods, potentially influencing the observed outcomes.

## 5. Conclusions

Investigating the predictive validity of the Bayley-III is of great importance for children, their parents, and clinicians in order to plan and implement specific treatments (if necessary). To conclude, our study has shown that, in this particular population, the cognitive scale of the Bayley-III may be used as a predictive tool for later motor skills, while we could not establish predictive validity for the motor and language scales. In terms of predicting school performance, the present findings indicate that the Bayley-III is not a reliable predictor. However, it is important to interpret these results with caution, as the sample size was small and the sample non-representative. This may limit the generalizability of the findings.

## Figures and Tables

**Table 1:** Descriptive statistics of the study samples.

		Sample 1 (*n* = 170)	Sample 2 (*n* = 69)
Sociodemographic characteristics			
Sex: Female	*n (%)*	77 (45%)	28 (41%)
Sex: Male	*n (%)*	93 (55%)	41 (59%)
SES: Low/medium	*n (%)*	97 (57%)	37 (54%)
SES: High	*n (%)*	73 (43%)	32 (46%)
Age at time t1	Mean (sd)	1.0 (0.11)	1.0 (0.13)
Age at time t2	Mean (sd)	6.4 (0.64)	8.9 (0.72)
Bayley-III			
Cognition: Normal/above average	*n (%)*	117 (78%)	52 (83%)
Cognition: Below average	*n (%)*	33 (22%)	11 (17%)
Language: Normal/above average	*n (%)*	95 (69%)	33 (60%)
Language: Below average	*n (%)*	42 (31%)	22 (40%)
Motor: Normal/above average	*n (%)*	107 (78%)	41 (82%)
Motor: Below average	*n (%)*	30 (22%)	9 (18%)
Motor skills			
Balancing backwards	Mean (sd)	0.05 (1.1)	
Sideways jumping	Mean (sd)	−0.35 (1.0)	
Standing long jump	Mean (sd)	0.03 (1.01)	
Pushups	Mean (sd)	−0.01 (1.04)	
Forward bends	Mean (sd)	−0.15 (1.22)	
School grades			
Math: High performance	*n (%)*		28 (41%)
Math: Low performance	*n (%)*		41 (59%)
German: High performance	*n (%)*		20 (29%)
German: Low performance	*n (%)*		49 (71%)
Physical Education: High performance	*n (%)*		15 (22%)
Physical Education: Low performance	*n (%)*		54 (78%)

Abbreviations: Bayley-III, Bayley Scales of Infant and Toddler Development 3rd edition; SD, standard deviation; SES, socioeconomic status; t1, time of Bayley-III assessment; t2, time of performed motor skills test (sample1) or time of information on school grades provided (sample 2).

**Table 2:** Associations (non-standardized regression coefficient + 95% confidence interval) between Bayley-III results and motor skills.

		Dependent Variable: Motor Skills				
Independent Variable: Below-Average Performance in the Respective Part of the Bayley-III		Balancing Backwards	Sideways Jumping	Standing Long Jump	Pushups	Forward Bends
Cognitive part	b	−0.45	−0.42	−0.54	−0.21	−0.59
	95% CI	(−0.9; −0.01)	(−0.81; −0.04)	(−0.95; −0.13)	(−0.71; 0.28)	(−1.05; −0.14)
	*p*	0.045	0.033	0.010	0.399	0.012
Language part	b	0.04	0.13	0.13	−0.20	0.12
	95% CI	(−0.37; 0.45)	(−0.23; 0.48)	(−0.29; 0.55)	(−0.66; 0.26)	(−0.37; 0.61)
	*p*	*p* = 0.860	*p* = 0.488	*p* = 0.527	*p* = 0.388	*p* = 0.624
Motor part	b	−0.38	−0.05	−0.12	−0.28	−0.05
	95% CI	(−0.83; 0.08)	(−0.46; 0.36)	(−0.57; 0.33)	(−0.79; 0.24)	(−0.58; 0.47)
	*p*	0.104	0.810	0.592	0.294	0.837

Abbreviations: b, non-standardized regression coefficient; 95% CI, 95% confidence interval. All associations were adjusted for age, sex, and SES.

**Table 3:** Associations (odds ratio + 95% confidence interval) between Bayley-III items and school performance.

		Dependent Variable: Low Performance in		
Independent Variable: Below-Average Performance in the Respective Part of the Bayley-III		Grade in Mathematics	Grade in German	Grade in Physical Education
Cognitive part	OR	1.22	1.84	2.13
	95% CI	(0.29; 5.07)	(0.34; 9.99)	(0.23; 20.02)
	*p*	0.782	0.481	0.51
Language part	OR	2.89	1.72	3.17
	95% CI	(0.8; 10.46)	(0.42; 7.08)	(0.48; 2.07)
	*p*	0.106	0.451	0.229
Motor part	OR	0.74	0.61	0.85
	95% CI	(0.14; 3.89)	(0.11; 3.39)	(0.12; 6.06)
	*p*	0.721	0.573	0.875

Abbreviations: OR, odds ratio; 95% CI, 95% confidence interval. All associations were adjusted for age, sex, and SES.

## Data Availability

Data collected in the LIFE Child study are not publicly available, as the publication of data is not covered by the informed consent provided by study participants. Because data sets contain potentially sensitive information, all researchers intending to access data are required to sign a project agreement. Researchers interested in accessing and analyzing data from the LIFE Child study may contact the data use and access committee (forschungsdaten@medizin.uni-leipzig.de) or TP (tanja.poulain@medizin.uni-leipzig.de).
